# Dataset of Vietnamese teachers’ perspectives and perceived support during the COVID-19 pandemic

**DOI:** 10.1016/j.dib.2020.105788

**Published:** 2020-05-29

**Authors:** Cam-Tu Vu, Anh-Duc Hoang, Van-Quan Than, Manh-Tuan Nguyen, Viet-Hung Dinh, Quynh-Anh Thi Le, Thu-Trang Thi Le, Hiep-Hung Pham, Yen-Chi Nguyen

**Affiliations:** a Institute of Theoretical and Applied Research, Duy Tan University, Hanoi 100000, Vietnam; b EdLab Asia Educational Research and Development Centre, Hanoi 100000, Vietnam; c Political Academy, Hanoi 100000, Vietnam; d Early Childhood Department, Hanoi National University of Education, Hanoi 100000, Vietnam; e University of Labour and Social Affairs, Hanoi 100000, Vietnam; f Hanoi University, Hanoi 100000, Vietnam; g Phu Xuan University, Hue 49000, Vietnam

**Keywords:** Education management, Teacher satisfaction, Teacher engagement, COVID-19, Vietnam

## Abstract

The COVID-19 pandemic has caused unprecedented damage to the educational system worldwide. Besides the measurable economic impacts in the short-term and long-term, there is intangible destruction within educational institutions. In particular, teachers – the most critical intellectual resources of any schools – have to face various types of financial, physical, and mental struggles due to COVID-19. To capture the current context of more than one million Vietnamese teachers during COVID-19, we distributed an e-survey to more than 2,500 randomly selected teachers from two major teacher communities on Facebook from 6th to 11th April 2020. From over 373 responses, we excluded the observations which violated our cross-check questions and retained 294 observations for further analysis. This dataset includes: (i) Demographics of participants; (ii) Teachers' perspectives regarding the operation of teaching activities during the pandemic; (iii) Teachers' received support from their schools, government bodies, other stakeholders such as teacher unions, and parents' associations; and (iv) teachers' evaluation of school readiness toward digital transformation. Further, the dataset was supplemented with an additional question on the teachers' primary source of professional development activities during the pandemic.

Specifications table

**Table:** 

Subject	Education, Education Management
Specific subject area	Education Management; School Effectiveness; Teacher Satisfaction
Type of data	Raw data in excel file and analyzed data
How data were acquired	Data was gathered using an online survey and converted into the .xlsx format for formal analysis in SPSS v.20.
Data format	Raw
	Analyzed
Parameters for data collection	The target population of this work is Vietnamese teachers whose teaching profession was affected by the COVID-19 pandemic. In light of the national school closures policy, almost every educational institution has to close until the end of April 2020. As a result, approximately one million teachers of various school types and educational levels were affected.
Description of data collection	An online survey has been delivered to 2,500 randomly selected Pre-K to post-secondary teachers. They are members of two major teacher communities on Facebook: MIE Expert Vietnam (38,600 members) and Vietnam Innovative Education Forum (14,000 members).
Data source location	Information is collected from secondary student institutes in Hanoi (Latitude 21°1′28.2"N, Longitude 105°50′28.21"E), Vietnam.
Data accessibility	Repository name: Harvard Dataverse
	Data identification number:
	Direct URL to data: https://doi.org/10.7910/DVN/FOCPKH,
	Harvard Dataverse, V1
	Repository Name: Mendeley
	Direct URL to data: https://data.mendeley.com/datasets/cy46h2rvwg/draft?a=d234e629-4509-4e7f-8379-e713efca803c

## Value of the data

• The dataset can be used for further analysis of teacher satisfaction and online teaching effectiveness with the focus on the chaotic context of a pandemic.
• The dataset can be used to construct models to evaluate educational leadership and school effectiveness in abnormal situations.
• The significant differences in Vietnamese teachers’ income before and during COVID-19 in this dataset can contribute to overall economic models on COVID-19’s damage.
• The dataset will be useful for school managers and policymakers to renovate policies, regulations, and practices to enhance teacher satisfaction, engagement, and effectiveness.
• The dataset presents a natural flow to measure teacher perceptions and satisfaction during COVID-19, which can be replicated in other countries.

## 1 Data Description

School effectiveness measurements include various factors related to students, teachers, and school managers that affect students’ academic achievement [1]. Although the Vietnamese government applied different systematic solutions to minimize the negative impacts of the COVID-19 pandemic [2], there is a lack of empirical evidence to support the decision-making process of school leaders. Under the chaotic circumstances caused by the pandemic, the significant shifts in learning and teaching habits require school leaders to face critical unknown-unknown issues. The formation of this dataset is an extension of our recent study on students’ learning habits during the pandemic [3,4], which contributes to the call of Elsevier on conducting research to tackle the current and potential impairments of the pandemic [5]. Regarding the sudden shift to online teaching and learning due to school closures, this dataset [6] portrayed Vietnamese teachers’ perspectives and teaching effectiveness during the pandemic and schools’ readiness toward the digital transformation.

Besides the information about the demographics of the participants, this dataset includes two primary groups of research items: (i) Teachers’ perceptions of factors associated with online teaching and learning; and (ii) Teachers’ opinions on school readiness and teaching effectiveness during the pandemic. The full questionnaires, variable code, and measurement parameters for all research items have been reposited in Harvard Dataverse [6]. Integrations among those variables can examine teacher satisfaction, self-reported teaching effectiveness, and school readiness during the pandemic. Tables 1, 2, 3

**Table 1:** Descriptive statistics of participant demographics.

Teacher satisfaction		N	Mean	Std. Deviation	Std. Error	Max	95% Confidence Interval for Mean		Min
							Lower Bound	Upper Bound	
Gender	Male	46	2.772	0.828	0.122	5	2.526	3.018	2
	Female	245	2.929	0.776	0.050	5	2.831	3.026	1
	Prefer not to disclose	3	2.167	1.041	0.601	3	-0.419	4.752	1
Exp	Less than 3 years	64	2.953	0.733	0.092	5	2.770	3.136	1
	From 3 to 5 years	48	2.823	0.796	0.115	4	2.592	3.054	1
	From 5 to 10 years	59	2.805	0.820	0.107	5	2.591	3.019	1
	More than 10 years	123	2.939	0.804	0.072	5	2.796	3.082	1
Degree	Diploma	13	2.615	0.506	0.140	3	2.309	2.921	2
	BA	181	2.909	0.759	0.056	5	2.797	3.020	1
	MA	89	2.888	0.878	0.093	5	2.703	3.073	1
	Doctor	11	3.091	0.801	0.241	4	2.553	3.629	2
Grade level	Pre-K	9	3.111	0.651	0.217	4	2.611	3.611	2
	Primary	100	2.825	0.783	0.078	5	2.670	2.980	1
	Lower Secondary	63	2.722	0.745	0.094	4	2.535	2.910	1
	Upper Secondary	66	3.068	0.784	0.096	5	2.875	3.261	1
	Post-Secondary	56	2.982	0.842	0.113	5	2.757	3.208	1
Subject	Sciences-related	87	2.948	0.743	0.080	5	2.790	3.107	1
	Social Sciences-related	70	2.971	0.751	0.090	5	2.792	3.151	1
	Foreign Language	57	2.763	0.835	0.111	5	2.542	2.985	1
	Others	80	2.869	0.837	0.094	5	2.682	3.055	1
School type	Public	191	2.901	0.747	0.054	5	2.794	3.007	1
	Private (normal)	49	3.041	0.822	0.117	5	2.805	3.277	1
	Private (bilingual/international)	37	2.730	0.838	0.138	4	2.450	3.009	1
	Continuing Education Center	13	2.615	0.820	0.228	4	2.120	3.111	1
	Others	4	3.375	1.493	0.747	5	0.999	5.751	2
Income before COVID-19 pandemic (USD)	<214	24	3.000	0.571	0.117	4	2.759	3.241	2
	214∼427	124	2.927	0.823	0.074	5	2.781	3.074	1
	427∼641	67	2.851	0.685	0.084	5	2.684	3.018	1
	641∼855	42	2.821	0.832	0.128	5	2.562	3.081	1
	>855	37	2.892	0.936	0.154	5	2.580	3.204	1
Income during COVID-19 pandemic (USD)	<214	100	2.905	0.695	0.070	4	2.767	3.043	1
	214∼427	133	2.868	0.790	0.069	5	2.733	3.004	1
	427∼641	35	3.071	0.768	0.130	5	2.807	3.335	2
	641∼855	19	2.684	1.157	0.265	5	2.126	3.242	1
	>855	7	3.000	1.000	0.378	5	2.075	3.925	2
Expected income after COVID-19 pandemic (USD)	<214	36	2.931	0.767	0.128	4	2.671	3.190	1
	214∼427	114	2.908	0.760	0.071	5	2.767	3.049	1
	427∼641	84	2.881	0.767	0.084	5	2.715	3.047	1
	641∼855	28	2.893	0.936	0.177	5	2.530	3.256	1
	>855	32	2.859	0.882	0.156	5	2.541	3.177	1
Total	294	2.896	0.789	0.046	5	2.806	2.987	1

**Table 2:** Descriptive statistics of teachers’ perceptions of factors that affect their teaching profession during COVID-19 pandemic.

	N	Range	Min	Max	Mean		Std. Deviation	Variance
					Statistic	Std. Error		
***COVID-19 pandemic is affecting teachers’…***								
Health (Feel_covid)	294	4	1	5	4.00	.049	.834	.696
Living habit (Feel_habit)	294	4	1	5	3.17	.045	.777	.604
Financial status (Feel_fin)	294	4	1	5	3.40	.052	.895	.801
***During COVID-19 pandemic, teachers received supports from…***								
School Board of Management (Sup_bod)	294	4	1	5	2.57	.065	1.114	1.242
Parents Association (Sup_parents)	294	4	1	5	2.15	.050	.865	.749
Teacher Union (Sup_union)	294	4	1	5	2.00	.048	.820	.672
Government (Sup_gov)	294	4	1	5	2.10	.051	.868	.754
Do not receive any support (Sup_none)	294	4	1	5	3.31	.072	1.227	1.505
***Regarding online teaching tools, teachers…***								
Mastered those ICT tools before COVID-19 pandemic (ICT_before)	294	4	1	5	3.25	.052	.884	.781
Do not face difficulty during COVID-19 pandemic (ICT_difficult)	294	4	1	5	3.24	.051	.871	.759
Know many types of online teaching tools (ICT_diverse)	294	4	1	5	3.50	.062	1.057	1.118
***Teachers often learn new ICT tools…***								
Proactively (ICT_proactive)	294	3	2	5	3.62	.047	.804	.647
More than what school provides (ICT_extend)	294	3	2	5	3.73	.045	.765	.585

**Table 3:** Descriptive statistics of teachers’ perceptions of school readiness, teaching effectiveness, and professional development during COVID-19 pandemic.

	N	Range	Min	Max	Mean		Std. Deviation	Variance
					Statistic	Std. Error		
***Regarding online teaching activities, teachers feel that …***								
It's as effective as normal class (Onl_effective)	294	4	1	5	2.96	.066	1.130	1.278
Students are more active (Onl_active)	294	4	1	5	3.04	.050	.860	.739
There is more workload (Onl_workload)	294	4	1	5	3.70	.051	.874	.763
They are more stressful (Onl_stress)	294	4	1	5	3.06	.051	.878	.771
***The school's readiness toward transformations during COVID-19 pandemic***								
ICT infrastructure (Ready_ICT)	294	4	1	5	3.35	.051	.872	.761
Teacher capabilities (Ready_teacher)	294	4	1	5	3.46	.050	.861	.741
Policies and regulation (Ready_policy)	294	4	1	5	3.40	.051	.875	.766
***During COVID-19 pandemic, teachers learnt new knowledge and skills on/due to…***								
ICT (New_ICT)	294	4	1	5	3.92	.042	.728	.530
Pedagogical (New_pedagogy)	294	4	1	5	3.64	.046	.787	.619
School's supportiveness (New_by_bod)	294	4	1	5	2.88	.053	.914	.836
Colleagues (New_by_colleagues)	294	4	1	5	3.02	.055	.936	.877
Do not have time to learn new things (New_lackoftime)	294	4	1	5	2.92	.060	1.021	1.042

## 2 Experimental Design, Materials, and Methods

The data was collected from 6th to 11th April 2020, the ninth week of national school suspension in Vietnam, due to the COVID-19 pandemic. Considering that there are more than one million teachers in Vietnam, it is impossible to reach all types of teachers across the country. Thus, the researchers focused on the two biggest teacher communities on Facebook: Microsoft Innovative Education Expert Vietnam - MIE (38,600 members) and Vietnam Innovative Education Forum – VIEF (14,000 members). Firstly, the survey was announced by the admins of those groups and attracted around 500 interactions from members. Additionally, we randomly selected 1,000 members from each group and sent them the survey URL, separately. Overall, a total of 373 responses was collected. Couples of cross-checking questions with reversed Linkert scales were embedded in the survey and helped us to eliminate 79 bias observations. Finally, we analyzed the dataset of 294 respondents.

The differences between teachers’ satisfaction among various demographic indicators and examined research items can be presented through ANOVA analysis. In particular, Table 4 shows the test of homogeneity of variances. Table 5 and Table 6 display the differences in teachers’ satisfaction among demographic indicators and teachers’ perception, respectively. The results of robust tests of equality of means are included in Table 7.

**Table 4:** Test of Homogeneity of Variances.

	Levene Statistic	df1	df2	Sig.
Gender	.976	2	291	.378
Exp	.887	3	290	.448
Degree	1.424	3	290	.236
Grade_level	.282	4	289	.889
Subject	.983	3	290	.401
School type	1.416	4	289	.229
Income before	2.102	4	289	.081
Income during	3.183	4	289	.014
Income expect	.582	4	289	.676
feel covid	.413	4	289	.800
Feel habit	.705	4	289	.589
feel fin	1.045	4	289	.384
Sup_bod	2.985	4	289	.019
Sup_parents	3.394^a^	3	289	.018
Sup_union	1.892	4	289	.112
Sup_gov	2.162	4	289	.073
Sup__none	4.093	4	289	.003
ICT_before	2.855	4	289	.024
ICT_difficult	.490	4	289	.743
ICT_diverse	2.128	4	289	.077
ICT_proactive	2.565	3	290	.055
ICT_extend	2.732	3	290	.044
Onl_effective	1.333	4	289	.258
Onl_active	5.001	4	289	.001
Onl_workload	.730	4	289	.572
Onl_stress	1.384	4	289	.239
Ready_ICT	4.785	4	289	.001
Ready_teacher	4.552	4	289	.001
Ready_policy	3.714	4	289	.006
New_ICT	2.163	4	289	.073
New_pedagogy	.214	4	289	.930
New_by_bod	7.690	4	289	.000
New_by_colleagues	9.253	4	289	.000
New_lackoftime	3.597	4	289	.007

**Table 5:** Differences in teachers’ satisfaction during COVID-19 pandemic among different demographics (ANOVA analysis).

Tearcher satisfaction		Sum of Squares	df	Mean Square	F	Sig.
Gender	Between Groups	2.566	2	1.283	2.074	.128
	Within Groups	180.020	291	.619		
Exp	Between Groups	1.181	3	.394	.629	.597
	Within Groups	181.405	290	.626		
Degree	Between Groups	1.478	3	.493	.789	.501
	Within Groups	181.108	290	.625		
Grade level	Between Groups	5.195	4	1.299	2.116	.079
	Within Groups	177.391	289	.614		
	Total	182.586	293			
Subject	Between Groups	1.701	3	.567	.909	.437
	Within Groups	180.885	290	.624		
School type	Between Groups	3.996	4	.999	1.617	.170
	Within Groups	178.590	289	.618		
Income before	Between Groups	.753	4	.188	.299	.878
	Within Groups	181.833	289	.629		
Income expect	Between Groups	.121	4	.030	.048	.996
	Within Groups	182.465	289	.631		
Total		182.586	293

**Table 6:** Differences in teachers’ satisfaction during COVID-19 pandemic among different examined perspectives (ANOVA analysis).

Tearcher satisfaction		Sum of Squares	df	Mean Square	F	Sig.
Feel_covid	Between Groups	7.589	4	1.897	3.133	.015
	Within Groups	174.997	289	.606		
Feel_habit	Between Groups	18.811	4	4.703	8.299	.000
	Within Groups	163.775	289	.567		
Feel_fin	Between Groups	11.009	4	2.752	4.636	.001
	Within Groups	171.577	289	.594		
Sup_union	Between Groups	26.275	4	6.569	12.145	.000
	Within Groups	156.310	289	.541		
Sup_gov	Between Groups	25.849	4	6.462	11.915	.000
	Within Groups	156.737	289	.542		
ICT_difficult	Between Groups	3.788	4	.947	1.531	.193
	Within Groups	178.798	289	.619		
ICT_diverse	Between Groups	2.524	4	.631	1.013	.401
	Within Groups	180.062	289	.623		
ICT_proactive	Between Groups	1.443	3	.481	.770	.512
	Within Groups	181.143	290	.625		
Onl_effective	Between Groups	5.463	4	1.366	2.228	.066
	Within Groups	177.123	289	.613		
Onl_workload	Between Groups	2.107	4	.527	.844	.498
	Within Groups	180.478	289	.624		
Onl_stress	Between Groups	5.803	4	1.451	2.372	.053
	Within Groups	176.783	289	.612		
New_ICT	Between Groups	7.422	4	1.856	3.061	.017
	Within Groups	175.164	289	.606		
New_pedagogy	Between Groups	8.382	4	2.095	3.476	.009
	Within Groups	174.204	289	.603		
Total		182.586	293

**Table 7:** Robust Tests of Equality of Means toward Teacher Satisfaction.

	Welch	Statistic*	df1	df2	Sig.
Income_during		.632	4	32.880	.643
Sup_bod		5.665	4	66.141	.001
Sup_parents⁎⁎					
Sup_none		7.515	4	85.241	.000
ICT_before		.911	4	25.462	.473
ICT_extend		2.066	3	53.860	.116
Onl_active		1.466	4	38.295	.231
Ready_ICT		6.891	4	25.282	.001
Ready_teacher		6.968	4	19.962	.001
Ready_policy		7.612	4	30.007	.000
New_by_bod		9.912	4	32.264	.000
New_by_colleagues		1.146	4	29.849	.354
New_lackoftime		5.489	4	56.265	.001

⁎ Asymptotically F distributed.

⁎⁎ Robust tests of equality of means cannot be performed for Tearcher satisfaction because at least one group has the sum of case weights less than or equal to 1.

Using questions with the five-points Linkert scale, this dataset demonstrated the factors associated with online teaching effectiveness, teacher satisfaction, and school effectiveness during the pandemic.

Regarding the control over online teaching effectiveness (ONL_EFF), we considered four factors. First, teachers’ overall perceptions of the impact of the pandemic (FEEL) are the aggregated result of the influence of the pandemic on their health; their living habits; and their financial status [7,8]. Second, we indicated the teachers’ received support (SUP) as a function of the support they receive from: School Board of Management; Parents Association; Teacher union; and Government bodies [9,10]. The question “I do not receive any support” was included to cross-check the validity of respondents. Third, teachers’ capability toward online teaching technologies (ICT_CAP) was the mean of their self-reported ICT (Information and Communication Technology) competency [10] before the pandemic emerged; the smooth of their online lesson during the pandemic; and the diversity of the tools which they mastered. Also, we added additional questions to examine the teacher's proactiveness in learning new ICT tools (ICT_ACT). We consider the influence of the above factors over online teaching effectiveness by the following regression:

ONL_EFF∼β0+β1*FEEL+β2*(SUP)+β3*(ICT_CAP)+β4*(ICT_ACT)+u

Regarding the influence over teacher satisfaction, we included teachers’ self-reports among the three following constructs [11]. First, teachers’ perceptions of online teaching activities (ONL_PER) were combined from the effectiveness of online class (in comparison with regular lessons – Onl_effective) [12], students’ activeness (Onl_active) [13], workload increment (Onl_workload), and level of stress during the pandemic (Onl_stress) [14]. During further analytical processes, the measurement scale of increased workload and degree of stress should be reversed to ensure the consistency of the overall construct. Second, the school's readiness toward digital transformations during the pandemic (READY) was indicated by the eagerness of ICT infrastructure, teacher capabilities, policies, and regulation [15]. Third, regarding professional development, we included types and sources of new know-how that teachers absorbed during the pandemic (PD). A cross-checking question was added to exclude invalid answers “I do not have time to learn new things.” If the response of this question is not consistent with the previous three, we will eliminate that observation. Considering teacher satisfaction as the primary outcome, the influence of those other factors listed above can be examined by the following regression:

SAT∼β0+β1*(ONL_PER)+β2*(READY)+β3*(PD)+u

## Declaration of Competing Interest

The authors declare that they have no known competing financial interests or personal relationships which have, or could be perceived to have, influenced the work reported in this article.

## Appendix Supplementary materials

Image, application 1

Image, application 2
