# Supplementary material for: Dataset of Vietnamese teachers’ perspectives and perceived support during the COVID-19 pandemic
Source: Data Brief. 2020 May 29;31:105788. doi: 10.1016/j.dib.2020.105788 (PMC7258812; doi:10.1016/j.dib.2020.105788)
Supplement: Supplementary file 1 [file mmc1.docx]

**Survey on Vietnamese teachers’ perspectives and perceived support during COVID-19**

| **Question** | **Code** | **1** | **2** | **3** | **4** | **5** |
| --- | --- | --- | --- | --- | --- | --- |
| 1. Your Gender | Gender | Male | Female | Prefer not to disclosure |  |  |
| 2. Teaching Experience | Exp | Less than 3 years | From 3 to 5 years | From 5 to 10 years | More than 10 years |  |
| 3. Teaching qualification | Degree | Diploma | BA | MA | Doctor |  |
| 4. You are teaching students at which grade level? | Grade_level | Pre-K | Primary | Lower Secondary | Upper Secondary | Post Secondary |
| 5. What subjects are you teaching? | Subject | Sciences-related | Social Sciences-related | Foreign Language | Others |  |
| 6. What type of school are you teaching? | School type | Public | Private (normal) | Private (bilingual/international) | Continuing Education Center | Other |
| 7. In overall, COVID-19 is affecting your health? | Feel_covid | Totally disagree | Disagree | Neither disagree nor agree | Agree | Totally agree |
| 7. COVID-19 changed your daily habit and make you tired? | Feel_habit | Totally disagree | Disagree | Neither disagree nor agree | Agree | Totally agree |
| 7. COVID-19 threatening your financial plan? | Feel_fin | Totally disagree | Disagree | Neither disagree nor agree | Agree | Totally agree |
| 8. Your monthly income before COVID-19? (USD) | Income before | <214 | 214~427 | 427~641 | 641~855 | >855 |
| 8. Your monthly income during COVID-19? (USD) | Income during | <214 | 214~427 | 427~641 | 641~855 | >855 |
| 9. What is your expected income after COVID-19? (USD) | Income expect | <214 | 214~427 | 427~641 | 641~855 | >855 |
| 10. During COVID-19, you received supports from school board of management? | Sup_bod | Totally disagree | Disagree | Neither disagree nor agree | Agree | Totally agree |
| 10. During COVID-19, you received supports from parents association? | Sup_parents | Totally disagree | Disagree | Neither disagree nor agree | Agree | Totally agree |
| 10. During COVID-19, you received supports from teacher union? | Sup_union | Totally disagree | Disagree | Neither disagree nor agree | Agree | Totally agree |
| 10. During COVID-19, you received supports from the government? | Sup_gov | Totally disagree | Disagree | Neither disagree nor agree | Agree | Totally agree |
| 10. During COVID-19, you do not receive any support? | Sup_none | Totally disagree | Disagree | Neither disagree nor agree | Agree | Totally agree |
| 11. I mastered online teaching tools since before COVID-19 | ICT_before | Totally disagree | Disagree | Neither disagree nor agree | Agree | Totally agree |
| 11. I do not face any difficult in online teaching during COVID-19 | ICT_difficult | Totally disagree | Disagree | Neither disagree nor agree | Agree | Totally agree |
| 11. I know many kinds of ICT platform, tools, and application to teach online | ICT_diverse | Totally disagree | Disagree | Neither disagree nor agree | Agree | Totally agree |
| 12. I often get to know the new technologies proactively | ICT_proactive | Totally disagree | Disagree | Neither disagree nor agree | Agree | Totally agree |
| 12. I know many tools and applications more than what my school provide | ICT_extend | Totally disagree | Disagree | Neither disagree nor agree | Agree | Totally agree |
| 13. I feel that online teaching is as effective as normal class | Onl_effective | Totally disagree | Disagree | Neither disagree nor agree | Agree | Totally agree |
| 13. I feel that students are actively engage with online sessions | Onl_active | Totally disagree | Disagree | Neither disagree nor agree | Agree | Totally agree |
| 13. I feel that the teaching workload is much more than before COVID-19 | Onl_workload | Totally disagree | Disagree | Neither disagree nor agree | Agree | Totally agree |
| 13. I feel stressful because of online teaching | Onl_stress | Totally disagree | Disagree | Neither disagree nor agree | Agree | Totally agree |
| 14. The ICT infrastructure of my school is ready for transformation during COVID-19 | Ready_ICT | Totally disagree | Disagree | Neither disagree nor agree | Agree | Totally agree |
| 14. The teacher capabilities of my school is ready for transformation during COVID-19 | Ready_teacher | Totally disagree | Disagree | Neither disagree nor agree | Agree | Totally agree |
| 14. The policies and regulations of my school is ready for transformation during COVID-19 | Ready_policy | Totally disagree | Disagree | Neither disagree nor agree | Agree | Totally agree |
| 15. During COVID-19, I have learnt lots of new ICT knowledge and skills | New_ICT | Totally disagree | Disagree | Neither disagree nor agree | Agree | Totally agree |
| 15. During COVID-19, I have learnt lots of new pedagogical knowledge and skills | New_Pedagogy | Totally disagree | Disagree | Neither disagree nor agree | Agree | Totally agree |
| 15. Most of my new knowledge and skill is due to the support of my school | New_by_bod | Totally disagree | Disagree | Neither disagree nor agree | Agree | Totally agree |
| 15. Most of my new knowledge and skill is due to the support of my colleagues | New_by_colleagues | Totally disagree | Disagree | Neither disagree nor agree | Agree | Totally agree |
| 15. I do not have proper time to elevate my profession | New_lackoftime | Totally disagree | Disagree | Neither disagree nor agree | Agree | Totally agree |
| 16. I am satisfying with online teaching and learning | Satis_teach_learn | Totally disagree | Disagree | Neither disagree nor agree | Agree | Totally agree |
| 16. I am satisfying with the supportiveness I received to ensure my living | Satis_life | Totally disagree | Disagree | Neither disagree nor agree | Agree | Totally agree |
